# Temporary Depletion of Microglia during the Early Postnatal Period Induces Lasting Sex-Dependent and Sex-Independent Effects on Behavior in Rats

**DOI:** 10.1523/ENEURO.0297-16.2016

**Published:** 2016-12-08

**Authors:** Jonathan W. VanRyzin, Stacey J. Yu, Miguel Perez-Pouchoulen, Margaret M. McCarthy

**Affiliations:** 1 Program in Neuroscience, University of Maryland School of Medicine, Baltimore, MD 21201; 2 Department of Pharmacology, University of Maryland School of Medicine, Baltimore, MD 21201

**Keywords:** behavior, liposomal clodronate, microglia, postnatal development

## Abstract

Microglia are the primary immune cells of the brain and function in multiple ways to facilitate proper brain development. However, our current understanding of how these cells influence the later expression of normal behaviors is lacking. Using the laboratory rat, we administered liposomal clodronate centrally to selectively deplete microglia in the developing postnatal brain. We then assessed a range of developmental, juvenile, and adult behaviors. Liposomal clodronate treatment on postnatal days 0, 2, and 4 depleted microglia with recovery by about 10 days of age and induced a hyperlocomotive phenotype, observable in the second postnatal week. Temporary microglia depletion also increased juvenile locomotion in the open field test and decreased anxiety-like behaviors in the open field and elevated plus maze. These same rats displayed reductions in predator odor–induced avoidance behavior, but increased their risk assessment behaviors compared with vehicle-treated controls. In adulthood, postnatal microglia depletion resulted in significant deficits in male-specific sex behaviors. Using factor analysis, we identified two underlying traits—behavioral disinhibition and locomotion—as being significantly altered by postnatal microglia depletion. These findings further implicate microglia as being critically important to the development of juvenile and adult behavior.

## Significance Statement

Microglia are critical regulators of postnatal brain development, a time important for the organization of neural architecture and the refinement of synaptic patterning. These immune cells engage in a range of functions, from promoting cell genesis and controlling cell number to facilitating the establishment and maturation of synaptic connections. Despite the current understanding of actions of microglia in the postnatal period, very little is known about how microglia’s influences on these early developmental processes affect the expression of behavior later in life. Our findings highlight the importance of microglia during this critical developmental window and suggest the necessity of these cells for the normal expression of juvenile and adult behavior.

## Introduction

Microglia, the brain’s primary immune cells, are derived from yolk-sac macrophage precursors that migrate into the brain during early embryonic development, where they take up permanent residence (Alliot et al., 1999; Ginhoux et al., 2010; Kierdorf et al., 2013). Throughout development, microglia continue to mature and multiply until they reach their adult numbers and effectively survey the brain (Davalos et al., 2005; Nimmerjahn et al., 2005; Schwarz et al., 2012; Nikodemova et al., 2015). Importantly, microglia mature and develop alongside neurons and astrocytes, placing these cells in a unique position to significantly influence the developmental trajectory of the brain.

In early brain development, microglia display an array of functions outside of their normal capacity to maintain homeostasis and respond to injury. Microglia regulate neural precursor populations and cell number by providing trophic support (Morgan et al., 2004; Antony et al., 2011; Ueno et al., 2013; Shigemoto-Mogami et al., 2014), phagocytose dead and dying cells, and actively induce cell death (Marín-Teva et al., 2004; Bessis et al., 2007; Wakselman et al., 2008; Cunningham et al., 2013), a process that continues even in the adult brain (Sierra et al., 2010). Microglia are critical to the proper formation of neural circuitry by “pruning” unnecessary synapses (Stevens et al., 2007; Schafer et al., 2012; Ji et al., 2013), aid in synaptic maturation and function (Tremblay et al., 2010; Paolicelli et al., 2011; Hoshiko et al., 2012; Squarzoni et al., 2014), and mediate the development of sex-specific circuitry (Lenz et al., 2013).

The neural control of normal behaviors also relies heavily on microglia function. Altering microglia function by constitutive genetic deletion of CX3CR1 results in deficient motor learning, spatial memory, and fear conditioning along with impairments in long-term potentiation in adult mice (Rogers et al., 2011). Moreover, these mice display abnormal social behaviors and increased repetitive and self-grooming behavior (Zhan et al., 2014). Genetic microglia depletion in juvenile and adult mice induces impairments in motor learning and memory and reduced fear conditioning (Parkhurst et al., 2013). Similarly, pharmacological attempts to specifically deplete microglia find abnormal spatial memory and social behavior in adult mice; however, behavioral deficits are no longer observed after microglia repopulate the brain (Torres et al., 2016).

These studies advance our understanding of the role microglia play in modulating behavior; however, the conclusions drawn are limited to the period of microglia dysfunction or depletion and do not directly address microglial influence specifically during development. It has been suggested that microglia are most critical for establishing brain architecture and circuitry necessary for normal behavior (Frost and Schafer, 2016), and little research has investigated the influence of microglia on the organization of brain and behavior during early periods of development.

To address this issue, we used liposomal clodronate to selectively deplete microglia during the first weeks of postnatal life, a time critical for the continued development of many brain regions, circuitry refinement, and sexual differentiation of the brain. Using male and female rats, we investigated two questions. First, does microglia depletion affect the postnatal developmental trajectory of pups? Second, are there enduring behavioral effects of temporary microglia depletion in juvenile and adult rats? We demonstrate pervasive sex-dependent and sex-independent effects of early postnatal microglia depletion on the later expression of behavior. Together, these data provide new insight into the role of microglia during the early postnatal period in mediating the normal maturation of behavioral circuits in the brain.

## Materials and Methods

### Animals

Adult Sprague-Dawley rats (Charles River Laboratories, Wilmington, MA) were housed in polycarbonate cages (20 × 40 × 20 cm) and maintained on a 12:12-h reverse light/dark cycle with *ad libitum* food and water. Animals were mated in our facility, and pregnant females were allowed to deliver naturally, with the day of birth designated as postnatal day 0 (PN0). On PN0, pups were sexed, treated, and culled to no more than 12 pups per dam. Both male and female pups were used in this study, with treatment groups and sexes balanced across four litters. All animal procedures were performed in accordance with the University of Maryland Baltimore animal care and use committee’s regulations.

### Microglia depletion

On PN0, 2, and 4, liposomal clodronate (LC; Encapsula NanoSciences, Brentwood, TN) or vehicle (VEH) liposomes were administered by bilateral intracerebroventricular (i.c.v.) injection, performed under cryoanesthesia. A 25-gauge 1-µL Hamilton syringe attached to a stereotaxic manipulator was placed 1 mm caudal to bregma and 1 mm lateral to the midline. The syringe was lowered 4 mm into the brain and backed out 1 mm. One microliter of drug or vehicle was infused over 30 s, and the procedure was repeated on the opposite hemisphere. The separation of pups from the dam was kept to a minimum, for a duration of approximately 1 h.

### Histology and immunohistochemistry

Rats of either sex were fatally anesthetized by intraperitoneal injection of Fatal Plus (Vortech Pharmaceuticals, Dearborn, MI) and transcardially perfused with PBS (0.1 m, pH 7.4) followed by 4% paraformaldehyde (PFA; 4% in PBS, pH 6.8). Brains were removed and postfixed in 4% PFA for 48 h at 4°C, then kept in 30% sucrose at 4°C until fully submerged. Coronal sections were cut into three alternating series at a thickness of 45 μm via cryostat (Leica CM3050S) and mounted on silane-coated slides.

Slide-mounted sections from one alternate series were rinsed in Tris-buffered saline (TBS; 0.05 m, pH 7.6) and incubated in 50% methanol with 0.3% hydrogen peroxide for 1 h at room temperature to inhibit endogenous peroxidase activity. Sections were rinsed with TBS again, blocked with 5% normal goat serum (NGS) in TBS + 0.4% Triton X-100 (TBS-T), and incubated overnight at room temperature in 5% NGS in TBS-T containing rabbit polyclonal antibody against ionized calcium binding adapter molecule 1 (Iba1; 1:1000 dilution; Wako Chemicals, Neuss, Germany; cat. #019-19741, RRID:AB_839504) to label microglia. Subsequently, sections were rinsed in TBS and incubated in 5% NGS in TBS-T containing biotinylated anti-rabbit secondary antibody (1:500 dilution; Vector Laboratories, Burlingame, CA; cat. #BA-1000, RRID:AB_2313606) for 1 h at room temperature, rinsed again in TBS, and incubated with ABC reagent (1:500 dilution; Vectastain Elite ABC Kit, Vector Laboratories; cat. #PK-6100) in TBS-T for 1 h at room temperature. After further rinsing in TBS, Iba1^+^ cells were visualized using nickel-enhanced DAB chromogen [0.05% 3,3′-diaminobenzidine, 0.2% nickel (II) sulfate, 0.006% hydrogen peroxide; all from Sigma-Aldrich, St. Louis, MO] in TBS for 1.5–3 min. Finally, sections were rinsed in TBS, counterstained with hematoxylin (Vector Laboratories; cat. #H-3401) according to the manufacturer’s protocol, cleared with ascending alcohol treatment, defatted in xylene, and coverslipped in DPX mounting medium.

### Image analysis and quantification

Immunolabeled sections were imaged using a Nikon Eclipse E600 microscope equipped with an MBF Bioscience CX9000 digital camera and analyzed using NIH ImageJ software. Modifications to image brightness and contrast were performed in ImageJ. For each brain region analyzed, four to six images were taken from two to three sections per rat. Regions of interest were drawn for each image, and the number of Iba1^+^ cells was counted. The Iba1^+^ cell number was normalized to the area of each region of interest to yield an Iba1^+^ cell density. Finally, the Iba1^+^ cell density was averaged across images to generate an average density value for each rat in each brain region. Image acquisition and analysis was performed by an experimenter blind to treatment condition.

### Behavioral testing

All behavioral testing was performed during the rat’s dark phase of the light cycle (∼2 h after the start of the dark phase) and under red light illumination. Behavioral tests were performed and scored offline (unless stated otherwise) by two experimenters blind to treatment condition.

### Developmental behavioral testing

Assessment of developmental behaviors began on PN5 and continued until PN15. Specific behaviors were assessed each day over the indicated age range for each rat pup.

#### Surface righting reflex (PN5–PN10)

Each pup was placed on its back on a flat surface. The time for the pup to return to its four limbs was recorded. The cutoff time for this test was 30 s.

#### Cliff aversion (PN5–PN10)

Each pup was placed on a flat surface with its snout and forepaws hanging over an edge. The time for the pup to completely retract from the edge was recorded, up to 30 s.

#### Wire suspension (PN10–PN14)

The pup’s forepaws were placed against a horizontal wire rod suspended above a padded bin. The duration of time each pup hung from the wire was recorded, up to 10 s.

#### Negative geotaxis (PN5–PN14)

Each pup was placed facedown on a ramp with a 25-degree incline, covered with a wire mesh to enable traction. The time for the pup to turn 180 degrees with its head and trunk oriented to the top of the incline was recorded, up to 30 s.

#### Locomotion (PN5–PN14)

Each pup was placed on a flat surface in the center of an underlaid circle (13-cm diameter). The time for all four legs of the pup to exit the circle completely was recorded, up to 30 s.

#### Nest seeking (PN5–PN15)

Rat pups were placed in the center of a rectangular arena (20 × 12 cm) between two goal containers filled with equal amounts of bedding, one containing fresh bedding and one containing bedding taken from their home nest. Four centimeters from either goal, the arena was marked to determine the “final side choice” of each pup. This measure was scored to account for pups that failed to reach either goal before the test duration expired. At the start of each trial, the pup was placed facing 90 degrees from either arm. The first orientation of the pup (toward or away from home bedding), the final side choice of the pup, and the goal container reached were recorded. Pup activity toward the nest bedding goal was given a positive score, whereas activity toward the clean bedding goal was given a negative score. Once the pup’s head and forepaws crossed into either goal (or reaching a cutoff time of 120 s), the pup was removed from the arena. Each pup was tested in two trials per day (intertrial interval of 30 s), and the pup’s starting position was counterbalanced between trials. The total score for each trial was calculated and averaged between the two trials, resulting in one score per pup, per day.

#### Maternal isolation-induced ultrasonic vocalization recording (PN8)

Ultrasonic vocalizations (USVs) were recorded for 3 min from rat pups that were isolated from maternal interaction. Ambient temperature was maintained at 22°C ± 1°C. On the day of testing, pups were placed individually into a sound-attenuated recording chamber (15 × 15 cm) and recorded for 3 min using an UltraSoundGate Condenser Microphone (CM16; Avisoft Bioacoustics, Glienicke, Germany), placed at a fixed height (10 cm) above the pups. The microphone was connected to a personal computer via an UltraSoundGate 116Hb recording interface (Avisoft Bioacoustics). Acoustic data were recorded at a sampling rate of 250,000 Hz in 16-bit format and analyzed using SASLab Pro (version 5.10; Avisoft Bioacoustics). Before analysis, recordings were processed with a fast Fourier transform (512 FFT, 75% frame size, flattop window, and 93.75% time window overlap). A high-pass filter was used to eliminate background noise below 10 kHz. USVs were automatically detected using a threshold-based algorithm (–35-dB threshold, 20-ms hold time) and confirmed by manual inspection to ensure that all calls were appropriately detected. Analysis was performed by an experimenter blind to the condition and sex of the pups.

### Juvenile behavioral testing

#### Open field test (PN25)

Rats were placed in an open polycarbonate arena (78 × 78 cm, 40 cm high) for 10 min. The floor was underlaid with a grid delineated into a center and perimeter regions. The time spent in the center grid squares and the number of grid crosses was recorded.

#### Spontaneous alternations (PN25–PN29)

Spontaneous alternation was tested using a black polycarbonate T-maze consisting of an entry arm (50 × 16 cm, 30 cm high) connected to a right and left arm (50 × 10 cm, 30 cm high). The right and left arms were split by a short divider that extended 10 cm into the entry arm to prevent the animal from simultaneously surveying both arms before entering. Rats were placed in the entry arm of the T-maze and allowed to explore until they completely entered either the right or left arm (sample phase). Upon entering an arm, a barrier was placed to confine rats to that arm for 30 s. Rats were removed and placed back into the entry arm and allowed to choose between the two arms again (test phase). The latency to enter an arm on the sample and test phases was recorded, as well as the chosen arm. Successful alternation was scored if the chosen test arm was opposite the chosen sample arm. Rats completed two trials (each trial comprised of a sample and test phase) on average each day (intertrial interval 10 min), and the total number of successful alternations was calculated as a percent of total number of trials (8–10 total trials per rat).

#### Novel object recognition (PN26–PN28)

On PN26, rats were placed into the same arena used for the open field test for 10 min to allow for continued habituation to the arena. On PN27, rats were exposed to a pair of identical objects (object A + A; sampling trial) placed on opposite ends of the arena for 5 min. After the initial exposure, animals were placed back in their home cages for 1 h, after which they were returned to the arena and exposed to the now familiar object and a novel object (object A + B). On PN28, animals were tested for their object memory 24 h after the first sampling trial, with a second novel object (object A + C). The time spent investigating each object was recorded, and the recognition index was calculated (time with novel object/time with novel object + time with familiar object). The position of objects in the arena and the order of object exposure was counterbalanced between treatment groups.

#### Social recognition (PN30)

Rats were singly housed in a test cage (identical to their home cage) for 2 h before the start of the test to allow for habituation. While singly housed, rats had *ad libitum* access to food and water. After 2 h, a stimulus rat (age PN15–21) was placed into the test cage with the test rat for 4 min. The stimulus rat was then removed, and the test rat remained in the test cage for a retention interval of 30 min. At this point, the original (now familiar) stimulus rat and a novel stimulus rat were placed in the test cage with the test rat. The time spent investigating the stimulus rat during the sample phase was recorded. During the recognition phase, the time spent investigating each of the stimulus rats was recorded, and recognition index was calculated (time with novel rat/time with novel rat + time with familiar rat).

#### Elevated plus maze (PN32)

Rats were placed in the center of a black polycarbonate elevated plus maze (elevated 72 cm from the ground) consisting of two opposing open (102.5 × 12 cm) and closed (102.5 × 12 cm, 45.5 cm high) arms and allowed to freely explore the maze for 5 min. The time spent in the open arms of the maze was recorded, and the percentage of the test duration spent in the open arms was calculated.

#### Predator odor exposure (PN33)

On PN32, rats were placed into the testing arena for 10 min to allow for habituation. The testing arena was divided into two compartments: a large main compartment (40 × 27 cm, 30 cm high) and a smaller rear compartment (20 × 27 cm, 30 cm high). An opaque polycarbonate barrier divided the two compartments but contained an opening (6 cm wide × 6 cm high) through which rats were able to pass. The purpose of this barrier was to provide an area in which the rats would be able to hide from the cat odor stimulus and allow for the expression of risk-assessing behaviors. Cat-scented pieces of cloth were prepared by placing the pieces in the bed of an adult male cat for 48 h before testing. On PN33, rats were tested in two consecutive phases: a baseline phase (2.5 min), during which the rats were exposed to an unscented piece of cloth (10 × 5 cm) folded in half lengthwise and placed in a large binder clip. The clip was placed at the far end of the large compartment (opposite the small compartment). At the end of the baseline phase, the unscented cloth square was replaced with a cat-scented cloth of similar dimensions, and the behavioral test continued for an additional 5 min. In both test phases, the time spent behind the barrier, duration and number of interactions with the cloth (defined as the touching of the cloth or binder clip with the nose or whiskers), duration and number of freezing bouts (defined as hunched posture with a complete absence of motion), duration and number of stretched-attending bouts (defined stretched, low-body posture with deliberate orientation and attention to the cloth square, while all locomotion is absent), and duration and number of stretched locomotor bouts (defined as stretched, low-body posture maintained while moving around the arena) were recorded. Data were normalized to a 5-min total duration for each phase of the test.

### Adult sex behavior testing

#### Female sex behavior (PN60+)

Intact and naturally cycling female rats were tested for the expression of female proceptive behaviors and lordosis in response to an actively mounting male rat. Females were tested daily throughout their estrus cycle for 20 min each day, and behaviors were scored for each rat on the day of pro-estrus (determined by vaginal cytology). Proceptive behaviors (number of hops and darts, solicitations) and lordoses were recorded, and a lordosis quotient was calculated (number of lordoses/number of successful mounts). Hops were defined as a short jump with the female landing on all four paws in a crouched position. Darts were defined as a short run with the female abruptly stopping in a crouched position. Solicitations were defined as the head-wise orientation toward the male followed by an abrupt runaway into a presenting posture. Behavior was scored live by an experimenter blind to the animal’s treatment.

#### Male sex behavior (PN60+)

Intact male rats were tested for male copulatory behaviors in response to a hormonally primed stimulus female rat. Males were tested once a week for 2 weeks in a 20-min behavioral test. Stimulus females were hormonally primed after being given 10 µg of estradiol benzoate in 0.1 ml sesame oil subcutaneously 1 and 2 days before testing, and 500 µg of progesterone in 0.1 ml sesame oil subcutaneously 6 h before testing. The number of mounts, intromission-like behaviors, and ejaculatory behaviors were recorded, as well as the latency to the first observation of each behavior. If none of these behaviors were observed, the latency was set equal to the test duration (1200 s). A mount was counted when the male mounted the female from the rear, placing both forepaws on the flanks of the female and thrusting against her. Intromission-like behaviors were counted when the male performed a mount, but during the thrusts quickly shifted his weight, engaged in extreme flexions of his haunches, and reflexively disengaged. Ejaculatory behaviors were defined as intromission-like behaviors that did not reflexively disengage, but instead the male remained in a hunched posture after thrusting. This was accompanied by a period of genital grooming followed by disinterest in the female. Behavior was scored live by an experimenter blind to the animal’s treatment.

### Statistical analysis

Statistical analysis was performed using R (R Core Team, 2016; version 3.2.1). All data were initially analyzed for a main effect of sex by two- or three-way ANOVA when appropriate. A main effect of sex was detected only for body weight. Therefore all other data were collapsed and analyzed independent of sex, with the exception of sex behavior, which was analyzed separately for males and females. Analysis proceeded using either Welch’s *t*-test or two-way ANOVA (repeated measures when age or test phase were a factor) when appropriate. In some cases, repeated-measures tests violated the assumption of sphericity; when this occurred, the Greenhouse–Geisser correction was applied (corrected *p* values are listed in Table 1). If a significant interaction was detected, *post hoc* pairwise comparisons were performed using the Holm’s sequential Bonferroni correction. Adjusted α values for *post hoc* tests are presented next to the calculated *p* value in Table 1. We chose *a priori* to analyze male sex behavior with a pairwise comparison on the second test day, as male performance improves with experience. The data for all male sex behaviors and female lordosis quotient did not follow a normal distribution and were therefore analyzed by a Wilcoxon rank sum test. Cohen’s *d*, η^2^, and Hodges–Lehmann (HL) estimates were calculated as measures of effect size for *t*-tests, ANOVAs, and Wilcoxon rank sum tests, respectively. Specific values for test statistics, *p* values, effect size, and power are listed in Table 1, and are referenced in text as superscript letters. Analyses were considered significant when *p* ≤ 0.05, unless otherwise noted in Table 1.

**Table 1.** Summary of statistical analyses.

Line	Data structure	Type of test	Description of analysis	Test value	*p*-value	Effect size	Power or 95% CI
a	Normal distribution	Welch’s *t*-test	PN5 hippocampus: VEH vs. LC	*t*_((2.9847)_ = 5.667	0.011	*d* = 4.625	0.984
b	Normal distribution	Welch's t-test	PN5 amygdala: VEH vs. LC	*t*_(3.7237)_ = 5.996	0.005	*d* = 2.38	0.596
c	Normal distribution	Welch's t-test	PN5 cortex: VEH vs. LC	*t*_(3.3363)_ = 2.915	0.054	*d* = 4.896	0.991
d	Normal distribution	Welch's t-test	PN5 hypothalamus: VEH vs. LC	*t*_(3.7531)_ = 2.108	0.059	*d* = 3.064	0.798
e	Normal distribution	Welch's t-test	PN10 hippocampus: VEH vs. LC	*t*_(1.0548)_ = –0.875	0.536	*d* = 1.045	0.13
f	Normal distribution	Welch's t-test	PN10 amygdala: VEH vs. LC	*t*_(2.6755)_ = –3.941	0.036	*d* = 2.461	0.457
g	Normal distribution	Welch's t-test	PN10 cortex: VEH vs. LC	*t*_(1.0034)_ = –2.013	0.293	*d* = 3.409	0.702
h	Normal distribution	Welch's t-test	PN10 hypothalamus: VEH vs. LC	*t*_(1.196)_ = –1.257	0.401	*d* = 1.42	0.197
i	Normal distribution	Welch's t-test	PN15 hippocampus: VEH vs. LC	*t*_(1.0067)_ = –1.248	0.429	*d* = 1.248	0.22
j	Normal distribution	Welch's t-test	PN15 amygdala: VEH vs. LC	*t*_(1.1786)_ = –1.437	0.36	*d* = 1.269	0.225
k	Normal distribution	Welch's t-test	PN15 cortex: VEH vs. LC	*t*_(1.312)_ = –1.269	0.384	*d* = 1.437	0.274
l	Normal distribution	Welch's t-test	PN15 hypothalamus: VEH vs. LC	*t*_(1.0299)_ = 0.254	0.841	*d* = 0.254	0.057
**Body weight**							
m	Normal distribution	3-way ANOVA	Main effect: sex	*F*_(1,29)_ = 236.343	<0.001	η^2^ = 0.068	1
**Female body weight**							
n	Normal distribution	2-way ANOVA	Main effect: treatment	*F*_(1,14)_ = 0.077	0.785	η^2^ = 0.072	0.058
o	Normal distribution	2-way ANOVA	Main effect: age	*F*_(1.146,16.037)_ = 617.607	<0.001	η^2^ = 0.974	1
p	Normal distribution	2-way ANOVA	Interaction: treatment × age	*F*_(1.146,16.07)_ = 2.842	0.108	η^2^ = 0.004	0.375
**Male body weight**							
q	Normal distribution	2-way ANOVA	Main effect: treatment	*F*_(1,15)_ = 6.045	0.027	η^2^ = 0.287	0.633
r	Normal distribution	2-way ANOVA	Main effect: age	*F*_(1.626,24.387)_ = 2222.172	<0.001	η^2^ = 0.992	1
s	Normal distribution	2-way ANOVA	Interaction: treatment × age	*F*_(1.626,24.387)_ = 2.019	0.161	η^2^ = 0.001	0.341
**Cliff aversion**							
t	Normal distribution	3-way ANOVA	Main effect: sex	*F*_(1,34)_ = 1.165	0.288	η^2^ = 0.033	0.183
u	Normal distribution	2-way ANOVA	Main effect: treatment	*F*_(1,36)_ = 0.381	0.541	η^2^ = 0.011	0.092
v	Normal distribution	2-way ANOVA	Main effect: age	*F*_(5,180)_ = 9.473	<0.001	η^2^ = 0.155	1
w	Normal distribution	2-way ANOVA	Interaction: treatment × age	*F*_(5,180)_ = 1.460	0.24	η^2^ = 0.031	0.263
**Surface righting**							
x	Normal distribution	3-way ANOVA	Main effect: sex	*F*_(1,34)_ = 0.177	0.676	η^2^ = 0.004	0.069
y	Normal distribution	2-way ANOVA	Main effect: treatment	*F*_(1,36)_ = 9.999	0.003	η^2^ = 0.217	0.868
z	Normal distribution	2-way ANOVA	Main effect: age	*F*_(5,180)_ = 3.973	0.014	η^2^ = 0.094	0.944
aa	Normal distribution	2-way ANOVA	Interaction: treatment × age	*F*_(5,180)_ = 2.134	0.11	η^2^ = 0.051	0.489
**Wire hang**							
bb	Normal distribution	3-way ANOVA	Main effect: sex	*F*_(1,34)_ = 0.934	0.34	η^2^ = 0.015	0.156
cc	Normal distribution	2-way ANOVA	Main effect: treatment	*F*_(1,36)_ = 25.742	<0.001	η^2^ = 0.083	0.999
dd	Normal distribution	2-way ANOVA	Main effect: age	*F*_(4,144)_ = 32.229	<0.001	η^2^ = 0.367	1
ee	Normal distribution	2-way ANOVA	Interaction: treatment × age	*F*_(4,144)_ = 2.223	0.087	η^2^ = 0.032	0.561
**Negative geotaxis**							
ff	Normal distribution	3-way ANOVA	Main effect: sex	*F*_(1,34)_ = 0.003	0.955	η^2^ < 0.000	0.05
gg	Normal distribution	2-way ANOVA	Main effect: treatment	*F*_(1,36)_ = 12.68	0.001	η^2^ = 0.024	0.934
hh	Normal distribution	2-way ANOVA	Main effect: age	*F*_(9,324)_ = 44.323	<0.001	η^2^ = 0.493	1
ii	Normal distribution	2-way ANOVA	Interaction: treatment × age	*F*_(9,324)_ = 1.228	0.291	η^2^ = 0.015	0.492
**Locomotion**							
jj	Normal distribution	3-way ANOVA	Main effect: sex	*F*_(1,34)_ = 1.109	0.3	η^2^ = 0.008	0.176
kk	Normal distribution	2-way ANOVA	Main effect: treatment	*F*_(1,36)_ = 93.876	<0.001	η^2^ = 0.723	1
ll	Normal distribution	2-way ANOVA	Main effect: age	*F*_(9,324)_ = 26.712	<0.001	η^2^ = 0.378	1
mm	Normal distribution	2-way ANOVA	Interaction: treatment × age	*F*_(9,324)_ = 7.873	<0.001	η^2^ = 0.088	1
nn	Normal distribution	Welch's t-test	Post hoc: PN5 LC vs. VEH	*t*_(29.15)_ = –0.398	0.694; α = 0.05	*d* = 0.125	0.066
oo	Normal distribution	Welch's t-test	Post hoc: PN6 LC vs. VEH	*t*_(34.126)_ = 0.766	0.449; α = 0.025	*d* = 0.25	0.117
pp	Normal distribution	Welch's t-test	Post hoc: PN7 LC vs. VEH	*t*_(24.031)_ = 1.869	0.074; α = 0.0125	*d* = 0.628	0.469
qq	Normal distribution	Welch's t-test	Post hoc: PN8 LC vs. VEH	*t*_(34.211)_ = 1.888	0.068; α = 0.01	*d* = 0.618	0.456
rr	Normal distribution	Welch's t-test	Post hoc: PN9 LC vs. VEH	*t*_(34.971)_ = 1.514	0.139; α = 0.01667	*d* = 0.494	0.315
ss	Normal distribution	Welch’s *t*-test	Post hoc: PN10 LC vs. VEH	*t*_(34.907)_ = 4.212	<0.001; α = 0.00714	*d* = 1.374	0.984
tt	Normal distribution	Welch’s *t*-test	Post hoc: PN11 LC vs. VEH	*t*_(35.873)_ = 4.639	<0.001; α = 0.0625	*d* = 1.503	0.994
uu	Normal distribution	Welch’s *t*-test	Post hoc: PN12 LC vs. VEH	*t*_(31.565)_ = 3.096	0.004; α = 0.0083	*d* = 1.021	0.864
vv	Normal distribution	Welch’s *t*-test	Post hoc: PN13 LC vs. VEH	*t*_(35.123)_ = 5.448	<0.001; α = 0.0056	*d* = 1.745	0.999
ww	Normal distribution	Welch’s *t*-test	Post hoc: PN14 LC vs. VEH	*t*_(35.301)_ = 8.084	<0.001; α = 0.005	*d* = 2.631	1
**Nest seeking total score**							
xx	Normal distribution	2-way ANOVA	Main effect: sex	*F*_(1,34)_ = 0.0002	0.989	η^2^ < 0.000	0.05
yy	Normal distribution	Welch’s *t*-test	VEH vs. LC	*t*_(34.052)_ = 3.927	<0.001	*d* = 1.285	0.97
**Nest seeking over time**							
zz	Normal distribution	3-way ANOVA	Main effect: sex	*F*_(1,34)_ = 0.015	0.904	η^2^ < 0.000	0.05
aaa	Normal distribution	2-way ANOVA	Main effect: treatment	*F*_(1,36)_ = 15.626	<0.001	η^2^ = 0.043	0.97
bbb	Normal distribution	2-way ANOVA	Main effect: age	*F*_(10,360)_ = 2.423	0.008	η^2^ = 0.053	0.943
ccc	Normal distribution	2-way ANOVA	Interaction: treatment × age	*F*_(10,360)_ = 1.087	0.371	η^2^ = 0.028	0.46
**Nest seeking latency**							
ddd	Normal distribution	3-way ANOVA	Main effect: sex	*F*_(1,34)_ = 0.278	0.602	η^2^ = 0.008	0.081
eee	Normal distribution	2-way ANOVA	Main effect: age	*F*_(10,360)_ = 47.962	<0.001	η^2^ = 0.458	1
fff	Normal distribution	2-way ANOVA	Main effect: treatment	*F*_(1,36)_ = 0.075	0.786	η^2^ = 0.002	0.058
ggg	Normal distribution	2-way ANOVA	Interaction: treatment × age	*F*_(10,360)_ = 0.369	0.847	η^2^ = 0.004	0.137
**Isolation-induced USVs**							
hhh	Normal distribution	2-way ANOVA	Main effect: sex	*F*_(1,23)_ = 0.253	0.62	η^2^ = 0.004	0.077
iii	Normal distribution	Welch’s *t*-test	VEH vs. LC	*t*_(23.928)_ = 7.337	<0.001	*d* = 2.795	0.999
**Spontaneous alternations**							
jjj	Normal distribution	2-way ANOVA	Main effect: sex	*F*_(1,34)_ = 1.802	0.188	η^2^ = 0.043	0.257
kkk	Normal distribution	Welch’s *t*-test	VEH vs. LC	*t*_(33.907)_ = 2.327	0.026	*d* = 0.762	0.626
**Social recognition index**							
lll	Normal distribution	2-way ANOVA	Main effect: sex	*F*_(1,25)_ = 0.299	0.59	η^2^ = 0.01	0.062
mmm	Normal distribution	Welch’s *t*-test	VEH vs. LC	*t*_(21.744)_ = 1.822	0.083	*d* = 0.702	0.441
nnn	Normal distribution	One sample *t*-test	VEH vs. 0.50	*t*_(15)_ = 3.599	0.003	*d* = 0.9	0.92
ooo	Normal distribution	One sample *t*-test	LC vs. 0.50	*t*_(12)_ = 0.224	0.827	*d* = 0.062	0.055
**Novel object: 1 h**							
ppp	Normal distribution	2-way ANOVA	Main effect: sex	*F*_(1,25)_ = 3.911	0.059	η^2^ = 0.135	0.477
qqq	Normal distribution	Welch’s *t*-test	VEH vs. LC	*t*_(21.868)_ = 0.119	0.907	*d* = 0.046	0.052
**Novel object: 24 h**							
rrr	Normal distribution	2-way ANOVA	Main effect: sex	*F*_(1,25)_ = 0.182	0.674	η^2^ = 0.006	0.069
sss	Normal distribution	Welch’s *t*-test	VEH vs. LC	*t*_(21.258)_ = –1.753	0.094	*d* = 0.677	0.416
**Open field center time**							
ttt	Normal distribution	2-way ANOVA	Main effect: sex	*F*_(1,34)_ = 0.061	0.806	η^2^ = 0.002	0.057
uuu	Normal distribution	Welch’s *t*-test	VEH vs. LC	*t*_(35.933)_ = –2.916	0.006	*d* = 0.944	0.807
**Open field grid crosses**							
vvv	Normal distribution	2-way ANOVA	Main effect: sex	*F*_(1,34)_ = 0.255	0.617	η^2^ = 0.003	0.078
www	Normal distribution	Welch’s *t*-test	VEH vs. LC	*t*_(25.858)_ = –7.427	<0.001	*d* = 2.484	1
**Elevated plus maze % open time**							
xxx	Normal distribution	2-way ANOVA	Main effect: sex	*F*_(1,34)_ = 1.612	0.213	η^2^ = 0.023	0.235
yyy	Normal distribution	Welch’s *t*-test	VEH vs. LC	*t*_(31.406)_ = –5.85	<0.001	*d* = 1.929	0.999
**PO time behind barrier**							
zzz	Normal distribution	3-way ANOVA	Main effect: sex	*F*_(1,34)_ = 0.0364	0.85	η^2^ = 0.001	0.054
aaaa	Normal distribution	2-way ANOVA	Main effect: treatment	*F*_(1,36)_ = 10.208	0.003	η^2^ = 0.158	0.875
bbbb	Normal distribution	2-way ANOVA	Main effect: test phase	*F*_(1,36)_ = 6.174	0.018	η^2^ = 0.04	0.677
cccc	Normal distribution	2-way ANOVA	Interaction: treatment × test phase	*F*_(1,36)_ = 1.701	0.201	η^2^ = 0.039	0.246
**PO freezing duration**							
dddd	Normal distribution	3-way ANOVA	Main effect: sex	*F*_(1,34)_ = 0.002	0.967	η^2^ < 0.000	0.05
eeee	Normal distribution	2-way ANOVA	Main effect: treatment	*F*_(1,36)_ = 3.616	0.065	η^2^ = 0.091	0.457
ffff	Normal distribution	2-way ANOVA	Main effect: test phase	*F*_(1,36)_ = 0.589	0.448	η^2^ = 0.016	0.116
gggg	Normal distribution	2-way ANOVA	Interaction: treatment × test phase	*F*_(1,36)_ = 0.955	0.335	η^2^ = 0.025	0.158
**PO stretch attend duration**							
hhhh	Normal distribution	3-way ANOVA	Main effect: sex	*F*_(1,34)_ = 0.0002	0.99	η^2^ < 0.000	0.05
iiii	Normal distribution	2-way ANOVA	Main effect: treatment	*F*_(1,36)_ = 9.223	0.004	η^2^ = 0.204	0.999
jjjj	Normal distribution	2-way ANOVA	Main effect: test phase	*F*_(1,36)_ = 27.268	<0.001	η^2^ = 0.389	0.999
kkkk	Normal distribution	2-way ANOVA	Interaction: treatment × test phase	*F*_(1,36)_ = 6.805	0.013	η^2^ = 0.097	0.719
llll	Normal distribution	Welch’s *t*-test	Post hoc: VEH baseline vs. odor	*t*_(19)_ = –4.95	<0.001; α = 0.0125	*d* = 1.459	0.994
mmmm	Normal distribution	Welch’s *t*-test	Post hoc: LC baselline vs. odor	*t*_(17)_ = –2.249	0.038; α = 0.025	*d* = 0.711	0.544
nnnn	Normal distribution	Welch’s *t*-test	Post hoc: baseline VEH vs. LC	*t*_(29.935)_ = 1.313	0.199; α = 0.05	*d* = 0.414	0.237
oooo	Normal distribution	Welch’s *t*-test	Post hoc: odor VEH vs. LC	*t*_(34.037)_ = 3.026	0.005; α = 0.01667	*d* = 0.965	0.824
**PO stretch locomotion duration**							
pppp	Normal distribution	3-way ANOVA	Main effect: sex	*F*_(1,34)_ = 0.032	0.86	η^2^ = 0.001	0.053
qqqq	Normal distribution	2-way ANOVA	Main effect: treatment	*F*_(1,36)_ = 11.985	0.001	η^2^ = 0.25	0.92
rrrr	Normal distribution	2-way ANOVA	Main effect: test phase	*F*_(1,36)_ = 8.605	0.006	η^2^ = 0.146	0.814
ssss	Normal distribution	2-way ANOVA	Interaction: treatment × test phase	*F*_(1,36)_ = 14.325	<0.001	η^2^ = 0.243	0.957
tttt	Normal distribution	Welch’s *t*-test	Post hoc: VEH baseline vs. odor	*t*_(19)_ = 0.846	0.408; α = 0.05	*d* = 0.209	0.099
uuuu	Normal distribution	Welch’s *t*-test	Post hoc: LC baselline vs. odor	*t*_(17)_ = –3.755	0.002; α = 0.01667	*d* = 0.739	0.577
vvvv	Normal distribution	Welch’s *t*-test	Post hoc: baseline VEH vs. LC	*t*_(27.927)_ = –1.521	0.14; α = 0.025	*d* = 0.506	0.329
wwww	Normal distribution	Welch’s *t*-test	Post hoc: odor VEH vs. LC	*t*_(20.041)_ = –4.225	<0.001; α = 0.0125	*d* = 1.435	0.99
**PO stimulus cloth approaches**							
xxxx	Normal distribution	3-way ANOVA	Main effect: sex	*F*_(1,34)_ = 0.032	0.86	η^2^ = 0.001	0.053
yyyy	Normal distribution	2-way ANOVA	Main effect: treatment	*F*_(1,36)_ = 8.312	0.007	η^2^ = 0.113	0.801
zzzz	Normal distribution	2-way ANOVA	Main effect: test phase	*F*_(1,36)_ = 19.22	<0.001	η^2^ = 0.135	0.989
aaaaa	Normal distribution	2-way ANOVA	Interaction: treatment × test phase	*F*_(1,36)_ = 1.116	0.298	η^2^ = 0.02	0.177
**PO stimulus cloth interaction duration**							
bbbbb	Normal distribution	3-way ANOVA	Main effect: sex	*F*_(1,34)_ = 0.067	0.798	η^2^ = 0.001	0.057
ccccc	Normal distribution	2-way ANOVA	Main effect: treatment	*F*_(1,36)_ = 26.650	<0.001	η^2^ = 0.34	0.999
ddddd	Normal distribution	2-way ANOVA	Main effect: test phase	*F*_(1,36)_ = 0.521	0.475	η^2^ = 0.014	0.108
eeeee	Normal distribution	2-way ANOVA	Interaction: treatment × test phase	*F*_(1,36)_ = 0.42	0.521	η^2^ = 0.011	0.097
**Male sex behavior**							
fffff	Non-normal	Wilcoxon rank sum test	Mount number: VEH vs. LC	*W* = 63	0.007	HL = 18.889	5.999 to 35.999
ggggg	Non-normal	Wilcoxon rank sum test	Mount latency: VEH vs. LC	*W* = 8.5	0.011	HL = -711.738	-1180 to -25.999
hhhhh	Non-normal	Wilcoxon rank sum test	Intromission number: VEH vs. LC	*W* = 57.5	0.028	HL = 9.0	4.33e-05 to 13.999
iiiii	Non-normal	Wilcoxon rank sum test	Intromission latency: VEH vs. LC	*W* = 8.5	0.009	HL = -862.834	-1167 to -121
jjjjj	Non-normal	Wilcoxon rank sum test	Ejaculation number: VEH vs. LC	*W* = 48.5	0.134	HL < 0.000	-6.933e-06 to 1
kkkkk	Non-normal	Wilcoxon rank sum test	Ejaculation latency: VEH vs. LC	*W* = 20	0.098	HL = -131.621	-663 to 5.161e-05
lllll	Normal distribution	Welch’s *t*-test	Hops and darts: VEH vs. LC	*t*_(16.893)_ = 0.532	0.602	*d* = 0.244	0.079
mmmmm	Normal distribution	Welch’s *t*-test	Solicitations: VEH vs. LC	*t*_(16.501)_ = 1.307	0.209	*d* = 0.602	0.236
nnnnn	Non-normal	Wilcoxon rank sum test	Lordosis quotient: VEH vs. LC	*W* = 45	1	HL < 0.000	-5.437e-06 to 2.74e-06
ooooo	Normal distribution	Welch’s *t*-test	Factor 1: male vs. female	*t*_(26.023)_ = –0.5	0.621	*d* = 0.187	0.077
ppppp	Normal distribution	Welch’s *t*-test	Factor 2: male vs. female	*t*_(18.074)_ = –0.691	0.499	*d* = 0.263	0.105
qqqqq	Normal distribution	Welch’s *t*-test	Factor 3: male vs. female	*t*_(20.761)_ = 0.704	0.49	*d* = 0.256	0.102
rrrrr	Normal distribution	Welch’s *t*-test	Factor 4: male vs. female	*t*_(26.982)_ = –0.572	0.572	*d* = 0.212	0.085
sssss	Normal distribution	Welch’s *t*-test	Factor 1: VEH vs. LC	*t*_(26.941)_ = –3.267	0.003	*d* = 1.187	0.865
ttttt	Normal distribution	Welch’s *t*-test	Factor 2: VEH vs. LC	*t*_(25.663)_ = –4.063	<0.001	*d* = 1.449	0.962
uuuuu	Normal distribution	Welch’s *t*-test	Factor 3: VEH vs. LC	*t*_(25.689)_ = 1.559	0.131	*d* = 0.556	0.301
vvvvv	Normal distribution	Welch’s *t*-test	Factor 4: VEH vs. LC	*t*_(21.724)_ = 1.415	0.171	*d* = 0.545	0.291

Behavioral data for factor analysis were normalized by log transformation before analysis. Only subjects that had complete behavioral datasets were included in the factor analysis (*n* = 16 for VEH, *n* = 13 for LC). Factor analysis was conducted with varimax rotation with a factor-loading cutoff of 0.3. The resulting factors were retained if their eigenvalue was greater than 1 (based on the Kaiser criterion), generating a four-factor solution for our dataset. Individual factor scores were calculated for each subject, and each factor was analyzed independently by Welch’s *t*-test.

## Results

### Liposomal clodronate treatment reduces microglia number and alters brain morphology

**Figure 1.**
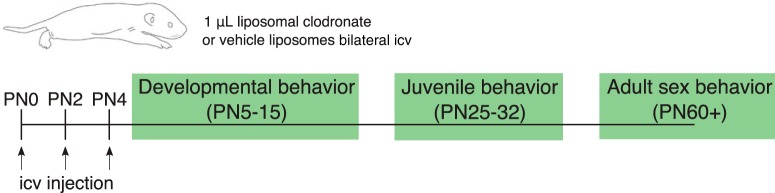
Treatment and behavioral testing timeline. Schematic depicting the ages at which treatments and behavioral testing were performed.

To determine the extent of microglia depletion and the time course of microglia recovery, we injected male and female rat pups i.c.v. with either LC or VEH liposomes on PN0, 2, and 4 (Fig. 1) and compared brain sections at PN5, 10, and 15. Gross morphological examination showed disruptions in overall brain morphology, most notably characterized by an enlargement of the ventricles and a thinning of the cortex. These effects were apparent as early as PN10 and highly variable between individual pups. In some cases, brain specimens were extremely fragile, making analysis from these individuals difficult. Examination of Nissl-stained sections revealed the presence of major brain regions that in extreme cases appeared displaced and compressed around the ventricles.

**Figure 2.**
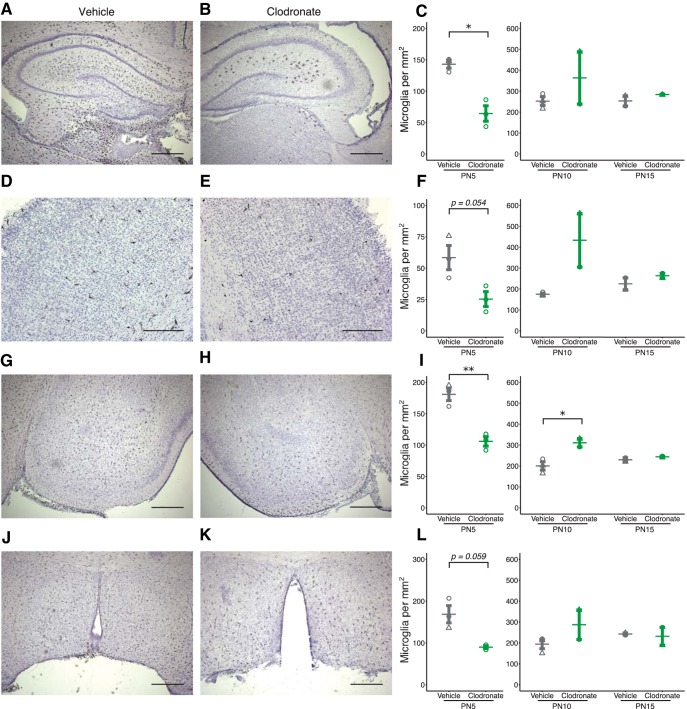
Microglia numbers are reduced after clodronate treatment and rapidly repopulate. Coronal sections of brains from PN5 VEH-treated (***a***, ***d***, ***g***, ***j***) and LC-treated (***b***, ***e***, ***h***, ***k***) rats. Representative images from sections labeled for Iba1 and counterstained with hematoxylin from the hippocampus (***a***, ***b***), cortex (***d***, ***e***), amygdala (***g***, ***h***), and hypothalamus (***j***, ***k***). Quantification of Iba1^+^ cell density in the hippocampus (***c***), cortex (***f***), amygdala (***i***), and hypothalamus (***l***) at PN5, 10, and 15. Scale bars = 500 μm (***a***, ***b***, ***g***, ***h***, ***j***, ***k***) or 250 μm (***d***, ***e***). Bars indicate group means ± SEM for VEH-treated (black) and LC-treated (green) rats of both sexes. Open circles indicate data from individual female rats, and triangles, from male rats. *p < 0.05, **p < 0.01. *n* = 2–3 per treatment.

We visualized microglia by immunohistochemistry using an antibody against the microglia/macrophage specific marker Iba1. Iba1^+^ cell density was quantified in various brain regions on PN5 (24 h after last LC injection). Analysis revealed significantly fewer Iba1^+^ cells in the hippocampus (Fig. 2*a–c*; *p* = 0.011^a^) and the amygdala (Fig. 2*g–i*; *p* = 0.005^b^), with a strong trend toward significant reduction in the cortex (Fig. 2*d–f*; *p* = 0.054^c^) and the hypothalamus (Fig. 2*j–l*; *p* = 0.059^d^). Overall, the effect of depletion ranged between 40% and 60% in the regions analyzed.

Limited quantification found that by PN10, Iba1^+^ cells from LC-treated pups robustly repopulated all regions of the brain^e–h^ (Fig. 2*c*, *f*, *i*, *l*). In some cases, this repopulation resulted in a greater number of microglia compared to VEH-treated controls (*p* = 0.036 for PN10 amygdala; Fig. 2*i*). The number of Iba1^+^ cells in LC-treated pups normalized by PN15, as sections from VEH- and LC-treated pups displayed comparable densities of Iba1^+^ cells across brain regions^i–l^ (Fig. 2*c*, *f*, *i*, *l*). Thus, the clodronate treatment paradigm resulted in a transient depletion of ∼50% of the microglia population throughout the brain during the first postnatal week, with rapid growth and repopulation that was complete by the end of the second postnatal week.

### Body weight throughout the lifespan

**Figure 3.**
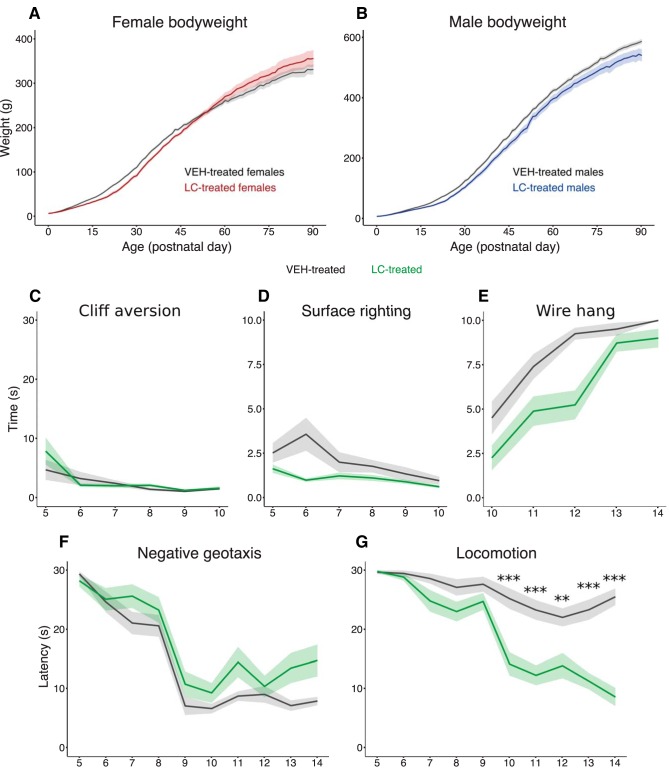
Microglia depletion alters the development of early reflexes, behaviors, and body weight throughout life. Body weight (in grams) in female (***a***) and male (***b***) rats measured daily from PN0 to PN90. ***c***, Time (in seconds) for rat pups to retreat from an edge (cliff aversion). ***d***, Time (in seconds) for rat pups to successfully right themselves (surface righting). ***e***, Duration (in seconds) rat pups hung from their forepaws before falling (wire hang). ***f***, Latency (in seconds) for rat pups to reverse orientation and face upward on an incline (negative geotaxis). ***g***, Latency (in seconds) for rat pups to leave a designated area (locomotion). Solid lines indicate group means for VEH-treated (black; ***a–g***) and LC-treated (red; ***a***) females, LC-treated males (blue; ***b***), and LC-treated rats of both sexes (green; ***c–g***). Shaded regions indicate ± SEM. **p < 0.01, ***p < 0.001. *n* = 18–20 per treatment.

We initially performed three-way repeated-measures ANOVA to examine the effects of sex and treatment on body weight throughout the animal’s life and found a significant effect of sex (*p* < 0.001^m^). This prompted us to repeat the analysis as two-way repeated-measures ANOVAs to assess the effect of treatment separately in male^q–s^ and female^n–p^ rats. Body weight increased with age for both male and female rats (*p* < 0.001 for females, Fig. 3*a*; *p* < 0.001 for males, Fig. 3*b*). Interestingly, LC-treated male rats consistently weighed less across the lifespan (main effect of treatment, *p* < 0.001), whereas LC-treated females did not differ in their weight compared to VEH-treated controls.

### Neonatal reflexes and behaviors

We did not detect an effect of sex on any of the developmental or juvenile behaviors examined, so we collapsed the data across sex and reanalyzed.

#### Cliff aversion

Latency to retreat for both VEH- and LC-treated pups improved with age (main effect of age, *p* < 0.001^t–w^; Fig. 3*c*).

#### Surface righting

LC-treated pups consistently righted themselves more quickly than VEH-treated controls (main effect of treatment, *p* = 0.003), and both groups improved with age (main effect of age, *p* = 0.014; Fig. 3*d*).^x–aa^

#### Wire hang

LC-treated pups hung for significantly less time than VEH-treated controls (main effect of treatment, *p* < 0.001; Fig. 3*e*), and hang time increased with age for all animals (main effect of age, *p* < 0.001).^bb–ee^

#### Negative geotaxis

LC-treated pups took longer to reorient themselves than VEH-treated controls (main effect of treatment, *p* = 0.001), and the latency to reorient decreased with age (main effect of age, *p* < 0.001; Fig. 3*f*).^ff–ii^

#### Locomotion

We detected a significant treatment × age interaction (*p* < 0.001; Fig. 3*g*). *Post hoc* comparisons found that LC-treated pups displayed increased locomotion as early as PN10 (*p* < 0.001), which persisted through PN14 (*p* < 0.001 for PN11, *p* = 0.004 for PN12, *p* < 0.001 for PN13, *p* < 0.001 for PN14).^jj–ww^

### Neonatal prosocial behaviors

#### Nest-seeking task

**Figure 4.**
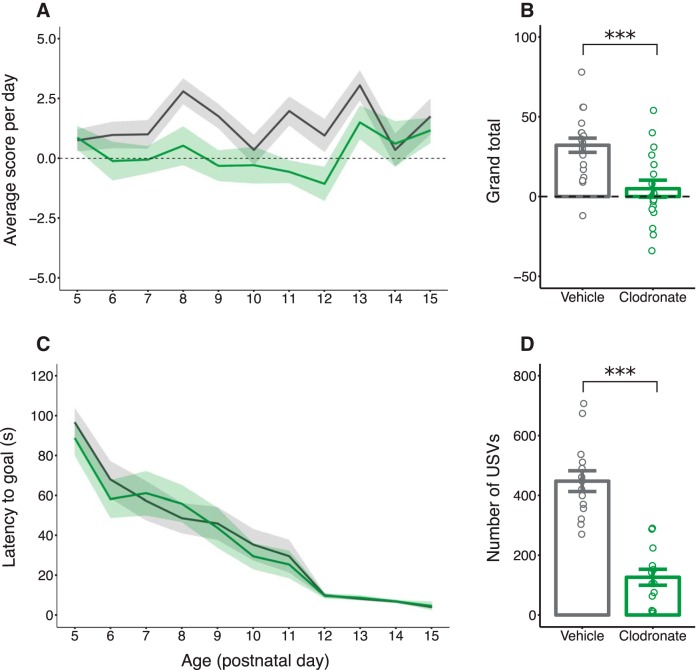
Postnatal microglia depletion impairs nest-seeking behavior and decreases USV emission. Quantification of data from the two-choice nest-seeking task represented as average score per day (***a***), average total score summed across all testing days (***b***), and average latency (in seconds) to reach either goal arm of the testing apparatus (***c***). ***d***, Quantification of the number of USVs produced by pups when separated from the mother on PN8. Solid lines and bars indicate group means for VEH-treated (black) and LC-treated (green) rats of both sexes. Shaded regions and error bars indicate ± SEM. Open circles represent data from individual rats. Horizontal dashed line indicates score equal to chance (***a***, ***b***). ***p < 0.001. *n* = 18–20 (***a–c***) and 13–14 (***d***) per treatment.

LC-treated pups performed significantly worse in the nest-seeking task, successfully locating and reaching the goal arm containing their home bedding fewer total times (*p* < 0.001; Fig. 4*b*). LC-treated pups reached the goal arm fewer times at all ages tested (main effect of treatment, *p* < 0.001; Fig. 4*a*), although the ability to seek the nest improved with age for both groups (main effect of age, *p* = 0.008). The latency to reach a goal arm significantly decreased with age regardless of treatment (main effect of age, *p* < 0.001; Fig. 4*c*).^xx–ggg^ Thus, it is unlikely that the observed impairments in performance were due to locomotor differences between groups.

#### Maternal isolation-induced USVs

LC-treated pups emitted significantly fewer USVs than VEH-treated controls when isolated from the dam (*p* < 0.001^hhh–iii^; Fig. 4*d*).

### Juvenile memory-dependent tasks

#### Spontaneous alternations

**Figure 5.**
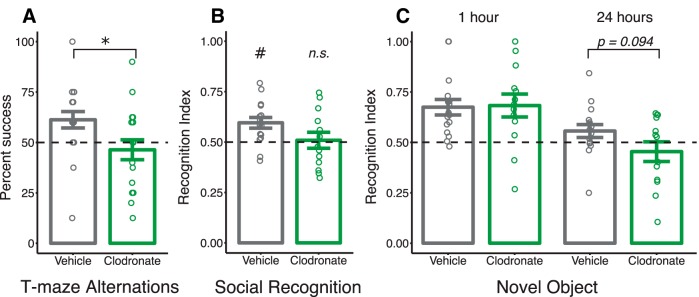
Postnatal microglia depletion induces selective memory impairment in juvenile rats. ***a***, Percentage successful spontaneous alternations in a T-maze from PN25 to PN29. ***b***, Social recognition index calculated on PN30 after a 30-min retention interval. ***c***, Novel object recognition index calculated after a 1-h (PN26) and 24-h (PN27) retention interval. Bars indicate group means ± SEM for VEH-treated (black) and LC-treated (green) rats of both sexes. Open circles represent data from individual rats. Horizontal dashed line indicates score equal to chance (***a–c***). *p < 0.05. #*p* < 0.01 compared with chance. *n* = 18–20 (***a***) and 13–16 (***b***, ***c***) per treatment.

LC-treated rats successfully alternated between arms significantly fewer times than VEH-treated rats (*p* = 0.026^jjj–kkk^; Fig. 5*a*).

#### Social recognition

VEH-treated rats displayed intact recognition memory, indicated by a recognition index significantly greater than chance (*p* = 0.003; 0.50 = chance level). LC-treated rats failed to recognize the familiar stimulus rat with a recognition index equal to chance (*p* = 0.863); however, direct comparison between groups did not reach statistical significance (*p* = 0.083; Fig. 5*b*).^lll–ooo^

#### Novel object recognition

Initial analysis found a strong trend for an effect of sex on novel object preference at 1 h (*p* = 0.059; data not shown), as males appeared to have an increased recognition index. The same analysis found no effect of sex at 24 h (*p* = 0.673; data not shown). After collapsing the data across sex, we found no difference between groups in recognition index at 1 h (*p* = 0.907; Fig. 5*c*), but did detect a trend toward a reduction in recognition index for LC-treated rats after a 24-h retention interval (*p* = 0.094).^ppp–sss^

### Juvenile locomotor and anxiety-like behavior

#### Open field

**Figure 6.**
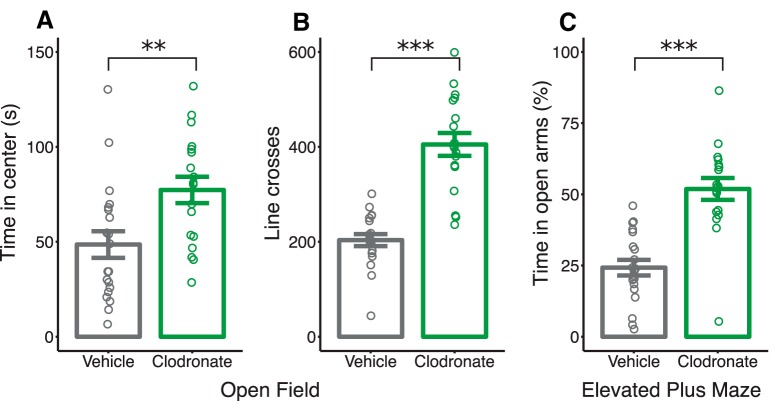
Postnatal microglia depletion increases locomotion, center time in the open field, and open arm time in the elevated plus maze in juvenile rats. Center time (***a***; in seconds) and gridline crosses (***b***) in an open field arena on PN25. ***c***, Percentage of time in the open arms of an elevated plus maze on PN32. Bars indicate group means ± SEM for VEH-treated (black) and LC-treated (green) rats of both sexes. Open circles represent data from individual rats. **p < 0.01, ***p < 0.001. *n* = 18–20 per treatment.

LC-treated rats displayed less anxiety-like behavior, spending significantly more time in the center of an open field (*p* = 0.006; Fig. 6*a*). Moreover, they exhibited a hyperlocomotive phenotype evidenced by an increased number of line crossings in the open field compared with VEH-treated rats (*p* < 0.001; Fig. 6*b*).^ttt–www^

#### Elevated plus maze

Similar to the open field test, LC-treated rats spent significantly more time in the open arms compared with VEH-treated controls (*p* < 0.001^xxx–yyy^; Fig. 6*c*).

### Juvenile fear, avoidance, and risk-assessing behaviors

**Figure 7.**
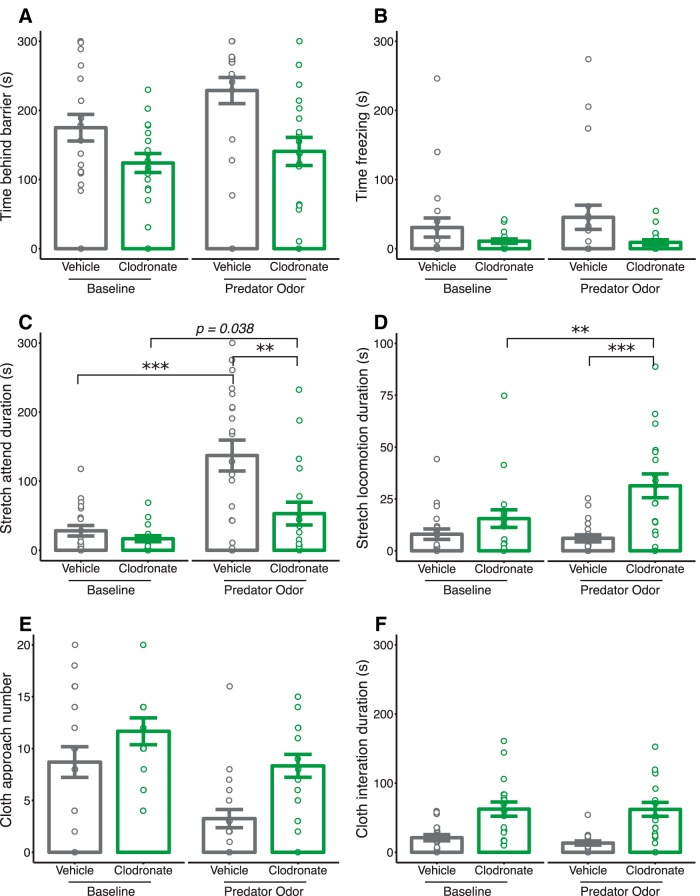
Microglia-depleted animals exhibit decreased fear and avoidance but display risk-assessing behaviors. Quantification of data from the predator odor exposure test (PN33) for time (in seconds) spent in various behaviors including time behind the barrier (***a***), freezing behavior (***b***), stretch-attend behavior (***c***), stretch-locomotion behavior (***d***), number of stimulus cloth approaches (***e***), and stimulus cloth interaction duration (***f***). Bars indicate group means ± SEM for VEH-treated (black) and LC-treated (green) rats of both sexes. Open circles represent data from individual rats. *p < 0.05, **p < 0.01, ***p < 0.001. *n* = 18–20 per treatment.

In the predator odor exposure test, LC-treated rats spent less time behind the barrier—a measure of behavioral avoidance—compared with VEH-treated controls (main effect of treatment, *p* = 0.002), and both groups spent more time behind the barrier in response to cat odor (main effect of test phase, *p* = 0.018; Fig. 7*a*). LC-treated rats appeared to spend less time freezing across test phases; however, this effect did not reach statistical significance (main effect of treatment, *p* = 0.065; Fig. 7*b*). We found treatment × test phase interactions for two measures of risk-assessing behaviors: stretch-attend duration (*p* = 0.013; Fig. 7*c*) and stretch-locomotion duration (*p* < 0.001; Fig. 7*d*). *Post hoc* analysis revealed that VEH-treated rats increased the amount of time engaged in stretch-attend behavior when exposed to cat odor (*p* < 0.001 for VEH), which was near significant for LC-treated rats (*p* = 0.038, α_adjusted_ = 0.025). LC-treated rats spent less time in stretched attendance than VEH-treated rats while exposed to cat odor (*p* = 0.005), with no baseline differences between groups. Interestingly, LC-treated rats spent more time engaged in stretched locomotion while exposed to cat odor (*p* = 0.002 compared to baseline), and significantly more compared with VEH-treated controls (*p* < 0.001), with no baseline differences between groups. Overall, LC-treated rats approached the stimulus cloth more than VEH-treated rats (main effect of treatment, *p* = 0.007), and the number of approaches decreased for both groups when the cloth was scented with cat odor (main effect of test phase, *p* < 0.001; Fig. 7*e*). Accordingly, LC-treated rats spent more time interacting with the stimulus cloth across both test phases (main effect of treatment, *p* < 0.001; Fig. 7*f*).^zzz–eeeee^

### Adult sex behavior

As microglia are critical to the masculinization of male sex behavior during the early postnatal period (Lenz et al., 2013), we analyzed adult male and female rats for the expression of sex behaviors. Accordingly, we predicted that LC-treated males would display impaired sex behavior, whereas female behavior would be unaffected by clodronate treatment.

#### Male sex behavior

**Figure 8.**
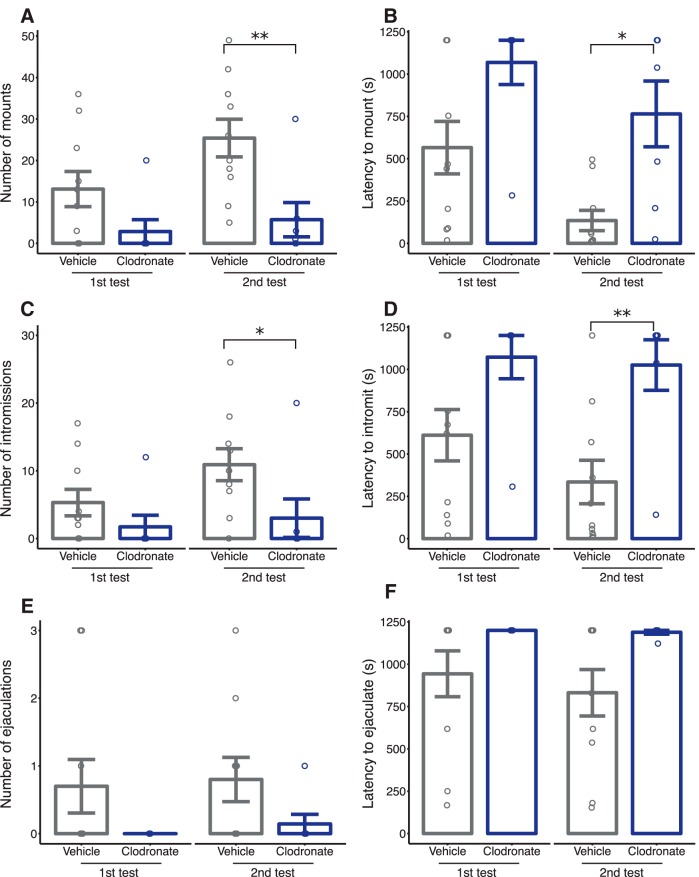
Postnatal microglia depletion induces deficits in adult male sex behaviors. Quantification of various components of male sex behavior including number of mounts (***a***), latency to first mount (***b***), number of intromissions (***c***), latency to first intromission (***d***), number of ejaculations (***e***), and latency to first ejaculation (***f***). Solid bars indicate group means ± SEM for VEH-treated (black) and LC-treated (blue) male rats. Open circles represent data from individual rats. *p < 0.05, **p < 0.01. *n* = 7–10 per treatment.

Consistent with our predictions, LC-treated males mounted fewer times than VEH-treated males on the second test day (*p* = 0.007; Fig. 8*a*). This corresponded to a significantly longer latency to first mount in LC-treated males (*p* = 0.011; Fig. 8*b*). Similarly, LC-treated males intromitted fewer times (*p* = 0.028; Fig. 8*c*), with an increased latency to first intromission (*p* = 0.009; Fig. 8*d*). We did not detect a difference in the expression of ejaculatory behaviors (*p* = 0.134; Fig. 8*e*) or the latency to first ejaculation (*p* = 0.098; Fig. 8*f*) between treatments, although this is likely due to the low incidence of ejaculation during our tests.^fffff–kkkkk^

#### Female sex behavior

**Figure 9.**
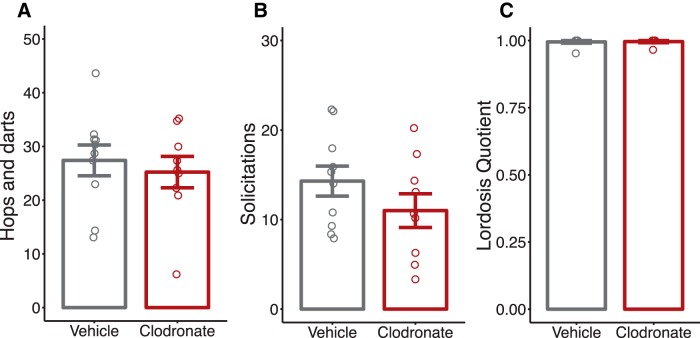
Adult female sex behavior is unchanged after postnatal microglia depletion. Quantification of female sexual proceptivity: number of hops and darts (***a***) and solicitations (***b***) and receptivity lordosis quotient (***c***). Solid bars indicate group means ± SEM for VEH-treated (black) and LC-treated (red) female rats. Open circles represent data from individual rats. *n* = 9–10 per treatment.

We found no difference in the expression of proceptive behaviors (hops and darts, *p* = 0.602, Fig. 9*a*; solicitations, *p* = 0.209, Fig. 9*b*) or receptivity as measured by the lordosis quotient (*p* = 1; Fig. 9*c*) between treatments.^lllll–nnnnn^

### Factor analysis of juvenile behaviors

**Figure 10.**
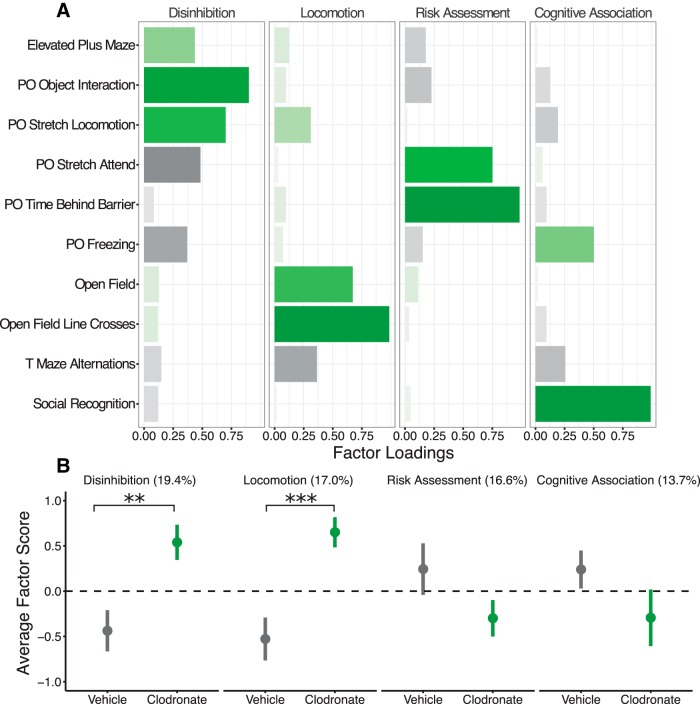
Factor analysis results. (***a***) Behavior factor loadings for each named factor. Longer, saturated bars indicate stronger loadings, with green indicating positive loading and black indicating negative loading. (***b***) Loading scores for VEH and LC rats plotted for each factor. The values in parentheses represent the percentage of total variance accounted for by each factor. Bars indicate group means ± SEM for VEH-treated (black) and LC-treated (green) rats of both sexes. **p < 0.01, ***p < 0.001. *n* = 13–16 per treatment.

We performed factor analysis on various outcomes from the juvenile behavioral battery to identify potential underlying behavioral constructs that might help explain the patterns of behavior we observed. Our analysis identified four factors to be sufficient to explain our data, accounting for 66.6% of total variance (Fig. 10*a*). The first factor (19.4% of total variance) contained positive loadings for performance on the elevated plus maze, predator odor object interaction duration, and stretch-locomotion and negative loadings for predator odor stretch attend duration and freezing duration. As the behaviors enriched in factor 1 largely reflected measures of behavioral disinhibition, we renamed it as such (“Disinhibition”). Similarly, the second factor (named “Locomotion”; 17.0% of total variance) contained positive loadings for open-field line crosses, open-field center time, predator odor stretch-locomotion, and negative loading for T-maze alternations, all behaviors reflecting locomotion and exploration. The third factor (“Risk Assessment”; 16.6% of total variance) included positive loadings for time behind the barrier and stretched attend duration in the predator odor test, and the fourth factor (“Cognitive Association”; 13.7% of variance) included positive loadings for predator odor freezing duration and social recognition index.

To determine whether these factors differed between sexes or treatment groups, we calculated factor scores for each subject. Comparisons between males and females found no significant differences for any of the four factors; however, comparisons between treatments found significantly higher scores for LC-treated rats in factor 1 (*p* = 0.003) and factor 2 (*p* < 0.001) compared with VEH-treated rats, with no differences in scores detected for either factor 3 or factor 4 (Fig. 10*b*).

## Discussion

Here we report lasting alterations in the expression of neonatal, juvenile, and adult behaviors after transient microglia depletion in the developing rat brain. These effects are apparent in early life, as both male and female rat pups displayed markedly deficient pro-social behaviors in addition to a pervasive hyperlocomotive phenotype. In juvenile animals, the consequences of early microglia depletion cross multiple behavioral domains and include working memory deficits and alterations in the expression of fear, anxiety-like, and risk-assessment behaviors. Adult rats show sex-specific sensitivity to early microglia depletion, exhibiting differential outcomes in body weight and male-specific impairments in sex behaviors.

The most remarkable of our findings is the immediate and lasting impact of transient microglia depletion on brain development and behavior. We observed perturbations in brain architecture as early as PN10, evidenced by the appearance of enlarged ventricles and a thinned cortex. These effects are likely attributable to microglia, as liposomal clodronate selectively depletes macrophages, via apoptosis, while sparing nonphagocytic cells (van Rooijen et al., 1996; van Rooijen and Hendrikx, 2010), and this technique has been used to deplete microglia without affecting other cell types (Faustino et al., 2011; Torres et al., 2016).

Similar alterations in gross morphology have been reported in CSF-1R knockout mice that display a >99% reduction in microglia throughout embryonic and postnatal development. Brain development in CSF-1R knockout mice appears to proceed normally during embryonic development, with abnormalities most prominent at 3 weeks of age (Erblich et al., 2011). Our findings suggest a much shorter window for the manifestation of these effects, as by PN10, rapid regrowth restored microglia numbers to levels comparable to controls. Moreover, 24 h after the last clodronate injection (PN5), we observed a ∼50% rate of depletion in many brain regions. It is especially striking, then, that modest reductions in microglia over a short period of time produce such profound brain abnormalities and major alterations in behavior. Perhaps equally notable, the rats in our study appeared to live healthy lives into adulthood, and the behavioral data had consistently small variance, similar to that of control rats despite large deviation in brain development.

Microglia are critical to the development of the embryonic and postnatal brain (Frost and Schafer 2016), yet data connecting early microglia function to behavioral maturation is currently lacking. One such study, published during the preparation of this manuscript, used a similar approach to address this issue (Nelson and Lenz, 2016). Our findings expand on those, as we used a behavioral battery to assess the appropriate acquisition and development of pups’ motor reflexes, strength, and coordination with the aim of identifying early markers of dysfunction. From this battery, we identified a hyperlocomotive phenotype that persisted through the juvenile period. Previous reports find no changes in locomotion with various microglia manipulations in adult mice (Rogers et al., 2011; Elmore et al., 2014; Torres et al., 2016); thus, it is likely that our findings reflect a novel developmental role for microglia in mediating the maturation of locomotor behavior.

The neonatal period of life also marks the development of early pro-social behaviors. We used a nest-seeking task and measured maternal isolation–induced USVs. The nest-seeking task measures pups’ ability to identify and locate the maternal nest and depends on the display of adequate olfactory, motor, and discriminatory abilities to discern nest bedding from clean bedding (Gregory and Pfaff, 1971), whereas pup USVs are instructional to the dam to elicit attention and retrieval. Together, these two behaviors represent two ethologically relevant and important aspects of the pup side of the maternal–pup relationship that are often disrupted in models of neurodevelopmental disorders (Branchi et al., 2001). Consistent with the notion of an early social deficit, microglia-depleted pups demonstrated no preference for the maternal bedding and emitted fewer isolation-induced USVs than their undepleted littermates. We did not determine whether maternal behavior toward pups was altered in those in which the microglia were depleted; additional studies will need to investigate the nature of mother–infant interactions as they pertain to the early, and perhaps later, social deficits reported here.

Others have reported effects of microglia depletion in mature mice on various memory-dependent tasks, social deficits, and conditioned fear responses. We find similar deficiencies across these behavioral domains in juvenile animals. Specifically, microglia-depleted rats had fewer successful spontaneous alternations, which may reflect impairments in working memory, but this response may also be interpreted as a broader measure of cognitive inflexibility or repetitive behavioral patterning. Despite these deficiencies, we did not find any difference in novel object preference, suggesting that microglia-depleted rats were capable of integrating and retaining information for prolonged periods of time.

Microglia-depleted rats also displayed impaired social memory. Taken together with our findings in the nest-seeking task, these data suggest pervasive disruptions in the processing of social information. Conspecific recognition is one aspect of a social interaction, and further studies are needed to examine whether the observed social impairments extend to more complex social behaviors. Along these lines, a recent report in rats found that neonatal microglia depletion induced minor deficits in juvenile play behavior and increased social avoidance in adulthood (Nelson and Lenz, 2016), consistent with our findings here.

There are conflicting reports on the role of microglia in anxiety-like behaviors (Rogers et al., 2011; Elmore et al., 2015). When assessing performance in the open field and elevated plus maze, we found that microglia-depleted juvenile rats exhibited a drastic reduction in anxiety-like behaviors. Given the hyperlocomotive phenotype in the open field, this may reflect a measure of behavioral disinhibition or impulsivity, rather than a measure of anxiety. To this point, in pilot studies and the data presented here, nearly 20% of microglia-depleted animals jumped off of the elevated plus maze during the test and continued to explore the testing room. One such animal ended the test in this manner in fewer than 5 s. Our findings are strikingly similar to those of Nelson and Lenz (2016), who found increased locomotion and decreased anxiety-like behavior in the open field and elevated plus maze, even observing comparable numbers of animals that jumped off the elevated plus maze open arms.

Rodents freeze in response to many fear-evoking stimuli. Others have reported reductions in freezing behavior in response to fear conditioning in mouse lines with genetic depletion of microglia (Rogers et al., 2011; Parkhurst et al., 2013). Fear conditioning requires the formation of a learned association between a particular cue (context or specific stimulus) and a negative outcome. To avoid the confounding effects of differences in learning, we used an assay to examine innate fear that involved a predator odor. We observed a decrease in avoidance behaviors in microglia-depleted juveniles and detected a strong trend toward a decrease in freezing behavior. As our testing arena contained an opaque barrier for the animals to retreat behind, it is likely that the magnitude of any freezing behavior was diminished and animals engaged in other defensive behaviors. Interestingly, microglia depletion induced opposite effects on measures of risk assessment. Microglia-depleted rats displayed more stretch-attend behavior and stretched locomotion toward the predator odor. These data together suggest that although the overt expression of fear and avoidance is decreased in microglia-depleted rats, they retain the ability to recognize and assess a potential threat and adjust their behavior accordingly. These findings are consistent with the notion of a behaviorally disinhibited phenotype, rather than a decrease in general anxiety-like behavior.

Whereas males and females were equally affected in our developmental battery and juvenile tests of memory, fear, and anxiety, the two sexes were differentially affected with respect to sexually dimorphic sex behaviors. To this point, we found male-specific deficits in the expression of sex behavior and not in the expression of female sex behavior. These findings corroborate those of Lenz et al., (2013), who found microglia to be a key mediator of the masculinization of sex behaviors. Our understanding of how microglia may mediate organizational sex differences in the brain is still in its infancy, but interestingly, many of the mechanisms by which sex differences are established are also developmental processes that are heavily influenced by microglia (Lenz and McCarthy, 2015; McCarthy et al., 2015). Further investigation into how microglia may act to sculpt sexually dimorphic circuitry in the brain is critical to advancing our understanding of a fundamental aspect of brain development.

A common theme throughout our behavioral data was the presence of a hyperlocomotive and behaviorally disinhibited phenotype observed in LC rats. Our factor analysis supports this conclusion, identifying four factors, two of which were significantly different between treatment groups. The first factor related to performance in the elevated plus maze, and stretch locomotion, stretch attend, cloth interactions, and freezing behavior in the predator odor assay. Together, this factor may reflect behavioral disinhibition across contexts. The second factor related to time in the center of an open field, open field grid crosses, predator odor stretch locomotion, and T maze alternations, which together may be representative of locomotion and exploratory behavior. We speculate that the effects of developmental microglia depletion affect the maturation of brain circuitry underlying these behavioral constructs. The exact cellular and circuit level manifestation of our findings remains a subject for further investigation.

## Synthesis

Reviewing Editor: Cheryl Sisk, Michigan State University Decisions are customarily a result of the Reviewing Editor and the peer reviewers coming together and discussing their recommendations until a consensus is reached. When revisions are invited, a fact-based synthesis statement explaining their decision and outlining what is needed to prepare a revision will be listed below. The following reviewer(s) agreed to reveal their identity: Anthony Auger, Staci Bilbo.

Two reviewers evaluated your manuscript and they agree that the study is well done, interesting, and makes a contribution to the field, especially in light of recent studies in mice suggesting that microglial depletion has little to no effect on brain and behavioral function. However, reviewers noted that significant effects of microglia on normal brain function most likely lie in development, and that is precisely what you have explored here. Reviewers also noted that you've described the impact of early-life microglial depletion on an impressive number of behavioral and phenotypic outcomes in male and female rats, and that the factor analysis method for representing all behavior data in one cohesive graph is particularly helpful to understand clearly which behaviors are preferentially affected compared to others when microglia are depleted. Reviewers offered several suggestions for clarification of certain aspects of the study. I agree with this positive review, and ask that you please address the questions/concerns below and make appropriate revisions.

1. Authors mention that at PN10, there was a difference in microglial morphology in vehicle-treated vs. LC-treated brains - however no data has been shown to support this claim. Further, it is not clear if this difference was seen in both sexes or only in one.

2. In case of microglial number quantification (Fig. 2) there are several concerns. Firstly, why is the "n" so low for certain groups, and can any conclusive inferences be drawn from such data? Next, it is important that the microglial cell number be displayed separately for males and females. This is important because it has been demonstrated that there is a sex difference in microglia number at baseline in certain regions of rat brain at P4 - the time point at which clodronate liposomes were administered to the rats (Schwarz et al, 2012).

3. One drawback of this study is the likelihood of non-specific toxicity of the clodronate. To confirm that this paradigm of LC-administration in neonatal pups does not lead to toxicity to other cell types, quantification of neurons, astrocytes and oligodendrocytes might be necessary.

4. Was there a specific frequency of USVs that was inhibited in LC-treated animals? It is known that specific frequencies of USVs stand for different behavioral aspects of the rodent, and a change in pattern in USVs can be indicative of a disease phenotype.

5. As the authors found hyperlocomotion in clodronate-treated animals, oligodendrocyte function may be altered as a result of microglial depletion. It might be worthwhile to look at oligodendrocyte number in both cerebrum and cerebellum.

Minor points:

1. The legends of several graphs are missing e.g. Y-axis on Fig 2 c,f,I,l and X-axis on Fig. 3 c-g.

2. Was the Iba1 cell density normalized to area/volume to account for slice-to-slice variability?

3. Perhaps table one can be moved into a supplementary figure, as the data are also presented in figure form.
